# Nanoemulsions as delivery systems for lipophilic nutraceuticals: strategies for improving their formulation, stability, functionality and bioavailability

**DOI:** 10.1007/s10068-019-00731-4

**Published:** 2020-07-23

**Authors:** Seung Jun Choi, David Julian McClements

**Affiliations:** 1 Department of Food Science and Technology, Seoul National University of Science and Technology, Seoul, 01811 Republic of Korea; 2 Departement of Interdisciplinary Bio IT Materials, Seoul National University of Science and Technology, Seoul, 01811 Republic of Korea; 3 Department of Food Science, University of Massachusetts, Amherst, MA 01003 USA; 4 Department of Food Science and Bioengineering, Zhejiang Gongshang University, Hangzhou, 310018 Zhejiang China

**Keywords:** Nanoemulsions, Bioavailability, Bioaccessibility, Stability, Delivery systems, Encapsulation

## Abstract

The food and beverage industry often need to encapsulate hydrophobic functional ingredients in their products, including colors, flavors, lipids, nutraceuticals preservatives, and vitamins. Encapsulation can improve the handling, water-dispersibility, chemically stability, and efficacy of these functional ingredients. In this review article, we focus on the design of nanoemulsion-based delivery systems to encapsulate, protect, and deliver non-polar bioactive agents, such as vitamin A, D and E, β-carotene, lycopene, lutein, curcumin, resveratrol, and coenzyme Q10. Initially, the challenges associated with incorporating these different bioactives into foods are highlighted. The relative merits and drawbacks of different nanoemulsion fabrication methods are then discussed. Finally, examples of the application of nanoemulsions for improving the stability and bioavailability of various kinds of hydrophobic vitamins and nutraceuticals are provided.

## Introduction

Emulsions are thermodynamically unstable colloidal dispersions that are usually formulated from two fluids that are immiscible with each other (typically oil and water in foods), with one of the fluids being dispersed as small spherical droplets in the other one (Friberg et al., 2004; McClements, 2015b). In this article, we focus on oil-in-water (O/W) emulsions, which consist of small oil droplets dispersed in water, since these are the type of emulsion most commonly used as a colloidal delivery system within the food industry. The precise size of the oil droplets within an O/W emulsion depends on the type and concentration of emulsifier and oil used, as well as on the homogenization method employed (McClements, 2015b). When an emulsion contains droplets with a mean diameter greater than 200 nm, it is usually categorized as a *conventional emulsion*. But when it contains droplets a mean diameter smaller than this value, it is sometimes referred to as a *nanoemulsion*. It should be noted, however, that there is currently no consensus on the upper limit for the mean droplet diameter defining a nanoemulsion. For instance, in different review articles on nanoemulsions, the upper limit has been proposed to be 100 nm (Dasgupta and Ranjan, 2018; Zhou et al., 2018), 200 nm (Mason et al., 2006; McClements and Rao, 2011), 500 nm (Gupta et al., 2016) or 1000 nm (Abramovits et al., 2010). This confusion is not surprising since there is no drastic change in all of the physicochemical or functional properties of emulsions (such as optical properties, creaming rate, rheology, or bioavailability) when one of these particle size limits is crossed. Instead, there tends to be a relatively gradual change that occurs at different particle sizes for different properties. For instance, a nanoemulsion may become almost optically transparent when the droplets have diameters less than around 50 nm, but become highly stable to creaming when the droplet size falls below 100 nm. In the current review, we assume that a nanoemulsion contains droplets with mean particles diameters between about 20–200 nm (Table 1).

**Table 1:** Comparison of the properties of conventional emulsions and nanoemulsions (Mason et al., 2006; McClements, 2011)

	Conventional emulsion	Nanoemulsion
Droplet diameter	> 200 nm	20–200 nm
Thermodynamic stability	Unstable	Unstable
Kinetic stability	Metastable	Metastable
Surface-to-mass ratio	0.07–70 m^2^/g	70–330 m^2^/g
Appearance	Turbid to opaque	Transparent to translucent

There has been growing interest in the application of nanoemulsions in food and beverage products over the past decade or so because, under some circumstances, they have potential advantages over conventional emulsions associated with their smaller droplet dimensions (McClements, 2011; McClements and Rao, 2011). Nanoemulsions exhibit greater stability to gravitational separation and droplet aggregation than conventional emulsions, which can lead to an extension in the shelf life of food and beverage products (Tadros et al., 2004). At their lower particle size range (< 50 nm), nanoemulsions are optically clear because the droplets they contain are so small that they do not scatter light strongly. This type of nanoemulsion can be used in products that need to be optically transparent, such as fortified waters and soft drinks. In some circumstances, nanoemulsions can be made to be much more viscous than conventional emulsions with the same oil content, due to their higher effective particle size or greater number of particle–particle interactions (McClements, 2011). This type of nanoemulsion can be used to create novel textural attributes in food and beverage products, or to reduce the number of calories they contain by creating low-fat viscous or semi-solid products. In addition to these advantages, nanoemulsions have also received considerable attention because they can be designed to increase the bioavailability of encapsulated hydrophobic bioactive agents, such as lipids, vitamins, or nutraceuticals (Acosta, 2009).

This article provides an overview of the current status of our understanding of the fabrication, physicochemical attributes, and functional applications of food-grade nanoemulsions. Oil-in-water nanoemulsions are the main focus of this article because they are the type most likely to be utilized within commercial food and beverage products.

## Nanoemulsion properties

The physicochemical and functional attributes of nanoemulsions depend on the nature of the droplets they contain. In this section, the most important characteristics of the droplets in nanoemulsions are therefore considered in order to facilitate a more rational design of efficacious nanoemulsion-based delivery systems.

### Droplet characteristics

#### Droplet size

The size of the droplets in a nanoemulsion is important because it impacts its physicochemical and functional properties, such as its appearance, texture, shelf-life, and gastrointestinal fate (McClements, 2010b; McClements, 2011). The droplet size distribution of an emulsion can be controlled by modulating homogenization conditions and/or system composition (Jafari et al., 2006; Schubert and Engel, 2004; Walstra, 1993). For instance, the intensity and duration of the energy used to break up the droplets during homogenization can be varied, as well as the type and level of oil and emulsifier used. As shown in Table 1, the difference in droplet size between nanoemulsions and conventional emulsions is one of the main reasons for the difference in their physicochemical and functional properties. The droplet size is usually expressed as the mean particle diameter, such as the volume moment mean (*d*_4,3_) or surface area moment mean (*d*_3,2_).

#### Droplet composition

The droplets within O/W nanoemulsions have a hydrophobic core comprising of oil molecules and a hydrophilic shell comprising of emulsifier molecules (McClements, 2015b). The emulsifier molecules orientate themselves so that their polar parts protrude into water, while the non-polar parts protrude into the oil droplet core. The hydrophobic core may be comprised of one or more non-polar ingredients, including digestible fats, fat substitutes, essential oils, oil-soluble vitamins, nutraceuticals, flavors, colors, and preservatives. The hydrophilic shell may be comprised of one or more amphiphilic or polar ingredients, including small-molecule surfactants, phospholipids, proteins, and polysaccharides. The type, amount, and conformation of surface-active components adsorbed to the droplet surfaces determines the thickness of the hydrophilic shell. Shell thicknesses in nanoemulsions have been estimated to be around 1–2 nm, 2–10 nm, and 10–50 nm for small molecule surfactants, protein monolayers, and biopolymer multilayers, respectively (McClements, 2011).

The thickness and composition of the interfacial layers may be fairly similar in nanoemulsions and conventional emulsions, but there is a large difference in the ratio of the shell-to-core dimensions. This ratio is much greater in nanoemulsions than in conventional emulsions, which means that the overall droplet composition and physicochemical properties of nanoemulsions is more sensitive to the nature of the materials present in the shell (Mason et al., 2006; Tadros et al., 2004). For instance, the refractive index and density of the droplets in nanoemulsions may be quite different from that in equivalent conventional emulsions, leading to appreciable differences in their optical properties and physical stability (McClements, 2011). The impact of oil core radius (*r*) and shell thickness (*δ*_s_) on the composition of the droplets in nanoemulsions can be estimated using the following expression:

1 $$\phi_{\text{s}} = \frac{{\left( {r + \delta_{\text{s}} } \right)^{3} - r^{3} }}{{\left( {r + \delta_{\text{s}} } \right)^{3} }}$$

Here, *ϕ*_s_ is the volume of the shell divided by the effective volume of the overall droplet (core + shell).

#### Droplet concentration

The droplet concentration in nanoemulsions is typically controlled by varying the ratio of oil-to-water used during their fabrication. It can also be manipulated by diluting or concentrating a nanoemulsion after it has been fabricated. Nanoemulsions and conventional emulsions prepared using the same oil content have the same oil phase volume fraction (*ϕ*). The effective disperse phase volume fraction (*ϕ*_e_) of nanoemulsions, however, may be quite different from that of conventional emulsions because of the small dimensions of the droplets they contain (Mason et al., 2006; Tadros et al., 2004). The value of *ϕ*_e_ can be estimated using the following expression (McClements and Rao, 2011):

2 $$\phi_{\text{e}} = \phi \times \left( {1 + \frac{{\delta_{\text{s}} }}{r}} \right)^{3}$$

**Fig. 1:**
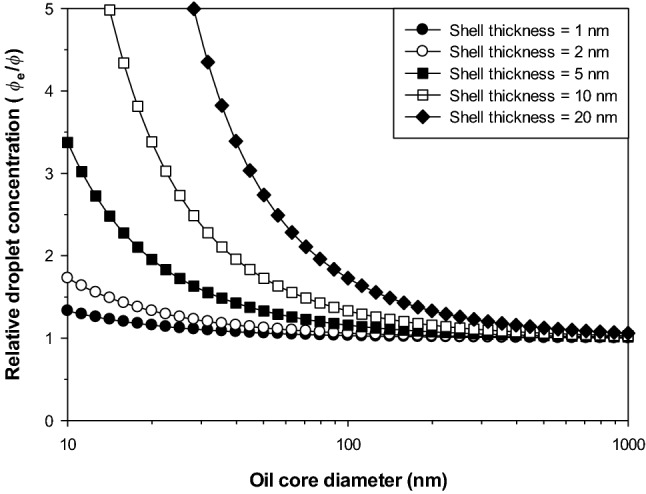
Predicted influence of the thickness of the emulsifier layer around oil droplets on the effective concentration of emulsifier-coated oil droplets in nanoemulsions

The radius of the oil core in conventional emulsions is much greater than the shell thickness (*r* ≫ δ_s_), which means that the effective disperse phase volume fraction is close to the oil phase volume fraction (*ϕ*_e_ = *ϕ*). Conversely, the effective droplet concentration of nanoemulsions may be considerably greater than their actual oil phase concentration because of their small droplet size (*r* ≈ δ_s_). This phenomenon may lead to the formation of nanoemulsions that have much higher viscosities than conventional emulsions at the same oil content. As shown by Eq. 2, the effective droplet concentration of nanoemulsions increases as the dimensions of the oil core decrease or the thickness of the shell increases (Fig. 1). Controlling the effective droplet concentration of nanoemulsions may be important to develop nanoemulsion-based food products with novel rheological properties (McClements, 2011).

#### Interfacial characteristics

The interfacial characteristics of the droplets in nanoemulsions play a major role in determining their physicochemical properties and functional attributes. The properties of nanoemulsions can therefore be manipulated by altering the composition, thickness, packing, rheology, charge, or environmental sensitivity of the interfacial layers. This can often be achieved by carefully controlling the type and concentration of the surface-active agents used to coat the oil droplets (Mason et al., 2006; McClements, 2015b). The interfacial characteristics are also influenced by changes in the conformation and interactions of the surface-active agents at the interface, as well as competitive adsorption processes (Dickinson, 2003).

The oil droplets in nanoemulsions have an electrical charge when they are coated by ionized surface-active substances, such as ionic surfactants, phospholipids, proteins, or polysaccharides (McClements, 2011). The electrical characteristics of the oil droplets impact the physical stability of nanoemulsions by altering the colloidal interactions acting between the droplets, which impacts their tendency to aggregate with each other. They also impact the chemical stability of nanoemulsions by altering the interaction of the oil droplets with other food components (such as antioxidants or prooxidants). Finally, they may modulate the gastrointestinal fate of nanoemulsions by altering their ability to stick to biological surfaces, their aggregation state in the human gut, or the ability of enzymes to adsorb to droplet surfaces.

The surface potential of the droplets in nanoemulsions is mainly determined by the electrical properties of the emulsifiers used to formulate them. When nanoemulsions are formed from emulsifiers with anionic groups (such as sulfate, carboxyl, or phosphate groups), the oil droplets tend to have a negative charge. Conversely, when they are formed from emulsifiers with cationic groups (such as amino groups), the oil droplets tend to have a positive charge. Most commercially available food-grade ionic surfactants and amphiphilic polysaccharides tend to have a negative charge, leading to anionic ionic droplets (Chanamai et al., 2002). The surface potential of oil droplets stabilized by proteins depends strongly on the pH of the surrounding aqueous phase (Gu et al., 2005). Droplets stabilized by proteins have a net positive charge when the pH is below the isoelectric point (pI) of the proteins, but have a net negative charge when the pH is higher than the pI. Protein-coated oil droplets have no net charge when the solution pH equals the pI, but there are patches of both negative and positive groups on their surfaces. The surface potential of the oil droplets in nanoemulsions can also be manipulated by adsorbing oppositely charged materials onto their surfaces after they have been formed (McClements, 2015b). For instance, the electrostatic deposition method has been used to create nano-laminated coatings around oil droplet surfaces by sequential adsorption of oppositely charged biopolymers (Johnston et al., 2006).

### Physicochemical properties of nanoemulsions

#### Optical properties

The scattering of light waves by nanoemulsions depends on the dimensions of the oil droplets (20–200 nm) compared to the wavelength of light (300–800 nm). When the droplets are much smaller than the wavelength of light (*d* < 50 nm), then they only scatter light very weakly and a nanoemulsion may appear transparent or translucent (Wooster et al., 2008). Conversely, when the droplets have dimensions that are fairly similar to the wavelength of light (100 < *d* < 200 nm), they scatter light strongly, and the nanoemulsions appear opaque. Carefully-designed nanoemulsions can therefore be used in applications where consumers desire clear or only slight turbid food and beverage products (McClements, 2010b). Besides droplet size, the optical properties of nanoemulsions are also affected by the relative refractive index and droplet concentration (Chantrapornchai et al., 1998; Chantrapornchai et al., 2001). Nanoemulsions tend to become clearer as the relative refractive index tends to unity, or the droplet concentration decreases. The particle size distribution also impacts the transparency/opacity of nanoemulsions. Highly polydisperse nanoemulsions may contain some large droplets that scatter light strongly making them appear more turbid (McClements and Rao, 2011).

#### Rheological properties

Emulsions exhibit a variety of rheological features depending on their compositions, structures, and interactions (Genevese et al., 2007; Mason et al., 2006; McClements, 2015b; Sonneville-Aubrun et al., 2004; Tadros et al., 2004). The small dimensions of the oil droplets in nanoemulsions can lead to quite different rheological attributes than conventional emulsions with the same oil phase volume fraction. Consequently, it is possible to modify the rheology of emulsion-based foods by reducing the droplet size. The rheological attributes of fluid nanoemulsions are usually characterized by their shear viscosity (*η*) (McClements, 2015b; Sonneville-Aubrun et al., 2004). For dilute nanoemulsions (< 5% droplets), the rheological properties can be predicted using Einstein’s equation:

3 $$\frac{\eta }{{\eta_{0} }} = 1 + 2.5 \times \phi$$

here, *η* is the shear viscosity of a nanoemulsion, *η*_0_ is the shear viscosity of the continuous phase, and *ϕ* is the volume fraction of the disperse phase. At higher droplet concentrations, the measured viscosity of fluid nanoemulsions is often appreciably greater than that predicted using Einstein’s equation due to the increased impact of droplet–droplet interactions. In this case, the viscosity can be predicted using the following semi-empirical equation:

4 $$\frac{\eta }{{\eta_{0} }} = \left( {1 - \frac{\phi }{{\phi_{\text{c}} }}} \right)^{ - 2}$$

**Fig. 2:**
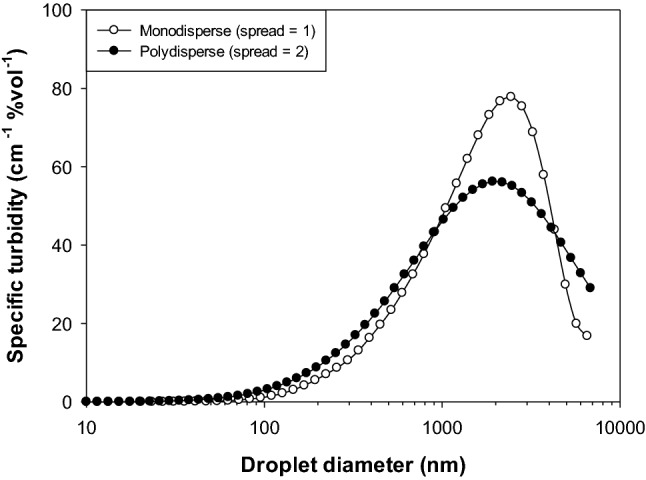
Predicted influence of the droplet size distribution of nanoemulsions with same mean droplet diameter on their specific turbidity

Here*, ϕ*_c_ is the critical disperse phase volume fraction above which the droplets are so closely packed together that they cannot flow past each other. As discussed earlier, for conventional emulsions *ϕ* can be assumed to be the same as the oil volume fraction because the interfacial layer has little effect. For nanoemulsions, however, *ϕ* has to be replaced by an effective droplet volume fraction (*ϕ*_e_), which is given by Eq. 2. The effective droplet volume fraction may be considerably greater than the oil volume fraction because of the relative high ratio of the shell layer thickness to oil core radius. Generally, the viscosity of an emulsion increases exponentially as the droplet concentration increases because the droplets become tightly packed together and cannot easily move past each other (Fig. 2). Under these conditions, the nanoemulsion exhibits solid-like characteristics, such as viscoelasticity and plasticity (McClements, 2015b; Quemada and Berli, 2002).

For nanoemulsions, the oil concentration where the shear viscosity increases steeply can be much lower than for conventional emulsions because the presence of the interfacial layer increases the effective droplet volume fraction. In principle, this phenomenon can be used to create food products that need to be highly viscous or gel-like with reduced fat contents.

#### Physical stability

Nanoemulsions are thermodynamically unstable and break down through various destabilization mechanisms, including gravitational separation, flocculation, coalescence, and Ostwald ripening (Friberg et al., 2004; McClements, 2015b). Nanoemulsions typically exhibit better stability to gravitational separation and droplet aggregation (flocculation and coalescence) than conventional emulsions, but worse stability to Ostwald ripening.

##### Gravitational separation

Gravitational separation is an important destabilization mechanism in most conventional emulsions because of the relatively large droplet size, and the density contrast between the oil and water phases. The density of edible oils is typically lower than that of water, which causes the oil droplets in O/W emulsions or nanoemulsions to move upwards, which is known as *creaming*. The velocity that an oil droplet moves upwards or downwards as a result of gravitational forces is given by Stokes’ law:

5 $$v_{Stokes} = - \frac{2}{9} \cdot \frac{{\left( {\rho_{2} - \rho_{1} } \right)}}{\eta } \cdot g \cdot r^{2}$$

**Fig. 3:**
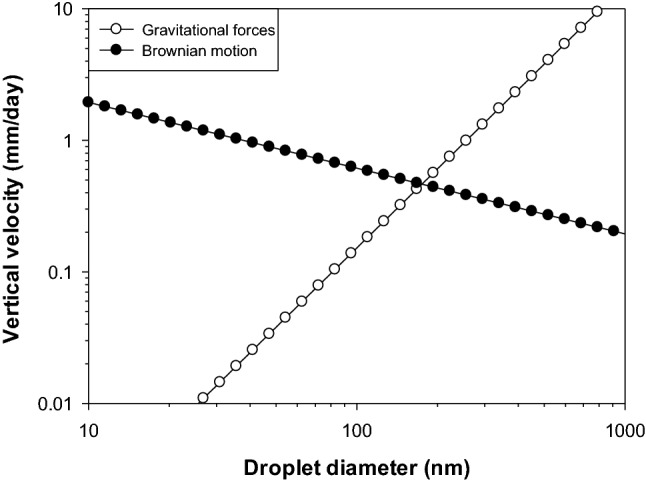
Predicted impact of gravitational forces and Brownian motion on the creaming velocity of differently sized oil droplets in an oil-in-water nanoemulsion. Typical values were used for the density of the oil phase (*ρ*_2_ = 920 kg/m^3^), the density of the water phase (*ρ*_1_ = 1000 kg/m^3^), and the viscosity of the water phase (*η*_1_ = 1 mPa s)

Here, *v*_Stokes_ is the creaming velocity, *r* is the droplet radius, *g* is the gravitational acceleration, *η* is the shear viscosity of the continuous phase, *ρ*_1_ is the density of the continuous phase, and *ρ*_2_ is the density of the oil phase. In reality, the droplets in nanoemulsions also move around as a result of Brownian motion, which is caused by the thermal energy of the system. Therefore, the overall velocity of an oil droplet depends on the balance between gravitational and Brownian motion forces that act upon it. As the droplet size increases, the tendency for droplets to move upwards due to gravitational forces increases, but the tendency to move around due to Brownian motion decreases (Fig. 3). As a result, the movement of droplets is dominated by gravity for large droplets but Brownian motion for small droplets (Mason et al., 2006).

In nanoemulsions, the nature of the interfacial layer may also impact the stability to gravitational separation. The emulsifiers in the interfacial layer usually have a higher density than both the oil and water phases. As a result, the net density of the droplets (core + shell) tends to increase as the thickness and packing of the interfacial layer increase. For nanoemulsions, this effect can be quite significant because the shell makes up an appreciable contribution to the overall volume of the droplets (Lee and McClements, 2010). As a result, this phenomenon can be utilized to match the density of the droplets to the aqueous phase, thereby inhibiting creaming or sedimentation.

##### Droplet aggregation

Another commonly observed instability mechanism in emulsions and nanoemulsions is droplet aggregation, i.e., flocculation, coalescence, or partial coalescence (McClements, 2015b). Flocculation is where two or more droplets cluster together but keep their individual identities. Coalescence is where two or more fluid droplets merge together and form a bigger droplet. Partial coalescence is where two or more partially crystalline droplets partially merge together to form a cluster. The colloidal interactions that act amongst droplets impact the stability of nanoemulsions to these destabilization mechanisms. Typically, when the attractive colloidal interactions outweigh the repulsive ones, the oil droplets tend to aggregate with each other. Conversely, when the repulsive colloidal interactions dominate, the droplets tend to be stable to aggregation. The most important attractive interactions are van der Waals and hydrophobic interactions, whereas the most important repulsive ones are steric and electrostatic interactions. Nanoemulsions are typically more stable to droplet aggregation than conventional emulsions because the magnitude of the attractive interactions decreases with decreasing droplet size (Tadros et al., 2004).

##### Ostwald ripening

This instability mechanism manifests itself as a progressive increase in the mean particle diameter of the droplets within an emulsion or nanoemulsion over time due, which is due to the net movement of disperse phase molecules from the smaller to the larger droplets (Kabalnov, 2001; Kabalnov and Shchukin, 1992). The thermodynamic driving force for this process is the increase in water-solubility of the oil phase as the droplets become smaller. As a result, there is a greater concentration of oil molecules dissolved within the water surrounding a small droplet than a large one. The resulting concentration gradient leads to the net movement of oil molecules from the smaller droplets to the larger droplets. Typically, the rate of Ostwald ripening is higher in a nanoemulsion than a conventional emulsion with the same composition. Ostwald ripening is only important in nanoemulsions that contain oils that have some solubility in water, such as flavor oils, essential oils, and short chain triglycerides. For these oils, it can be inhibited by mixing them with another oil that has got an extremely low solubility in water (such as a long chain triglyceride or mineral oil).

#### Chemical stability

There may be appreciable differences in the chemical stability of nanoemulsions and conventional emulsions because of the differences in the droplet dimensions. In particular, the specific oil–water interfacial area increases as the droplet size decreases, which can expose more of a chemically labile oil to reactive substances in the surrounding aqueous phase. As an example, lipid oxidation in emulsions mainly occurs at the oil droplet surfaces due to the presence of water-soluble prooxidants such as transition metals (Berton-Carabin et al., 2014; Lee et al., 2019; Waraho et al., 2011). As a result, lipid oxidation would be expected to occur faster in nanoemulsions than conventional emulsions. This hypothesis is supported by the results of Lee et al. (2011), which showed that lipid oxidation was faster in nanoemulsions (*d*_4,3_ ≈ 66 nm) than in conventional emulsions (*d*_4,3_ ≈ 325 nm), which were both stabilized by whey protein. Research conducted by Lethuaut et al. (2002) also supports this hypothesis, where it was shown that lipid oxidation decreased with increasing droplet size (*d*_4,3_ ≈ 0.5, 1.9, and 27.0 μm).

Many hydrophobic functional ingredients that are encapsulated in emulsions or nanoemulsions can be chemically degraded by oxygen, heat, light, and peroxides. For instance, nutraceuticals such as carotenoids, curcumin, and polyphenols are all susceptible to chemical degradation. The chemical stability of these compounds may, therefore, be influenced by the size of the droplets in an emulsion or nanoemulsion. For instance, it has been shown that curcumin is more stable when encapsulated in larger droplets than in smaller ones, which was attributed to the lower oil–water interfacial area of the conventional emulsions (Zou et al., 2015). In addition, the penetration of light waves (UV and visible) into a nanoemulsion depends on the oil droplet size since this impacts the light scattering profile. As a result, the chemical stability of encapsulated photo-sensitive functional ingredients may be different in nanoemulsions and emulsions.

It should be noted, however, that the impact of droplet size on the chemical degradation of emulsified oils and encapsulated hydrophobic bioactive agents has not been observed in all studies. Indeed, some researchers have claimed that droplet size only plays a minor role in controlling the chemical stability of emulsions and nanoemulsions, and that emulsifier type, emulsifier location, oil phase composition, and homogenization methods play a more important role in controlling lipid oxidation in many systems (Berton-Carabin et al., 2014; Osborn and Akoh, 2004; Walker et al., 2015).

## Food-grade nanoemulsion preparation

Nanoemulsions can be fabricated using a variety of approaches. They can be fabricated using some of the same approaches employed to produce conventional emulsions, as well as by some methods that are unique to nanoemulsions. The methods employed for nanoemulsion formation can be conveniently categorized as either high- or low-energy approaches (Acosta, 2009; Anton and Vandamme, 2009; Leong et al., 2009; Tadros et al., 2004; Yang et al., 2012).

High-energy approaches employ mechanical devices, such as high-pressure homogenizers, microfluidizers, or ultrasonicators, to generate powerful disruptive forces that mix and disrupt the oil and water phases leading to the formation of small droplets (Gutiérrez et al., 2008; Leong et al., 2009; Velikov and Pelan, 2008; Wooster et al., 2008). Mechanical devices are typically the most popular approach for creating nanoemulsions in the food industry because they are widely available, easy to scale-up, and have a high throughput (McClements and Rao, 2011). Another advantage of these methods is that nanoemulsions can be fabricated from a diverse array of oils and emulsifiers, starting materials with a range of different viscosities can be used, and the particle size can be controlled more easily.

Low-energy approaches do not require the utilization of mechanical devices to form nanoemulsions. Instead, the composition and/or environmental conditions of a surfactant–oil–water mixture are altered in a specific way, which leads to the generation of a nanoemulsion. Low-energy approaches include spontaneous emulsification, phase inversion temperature, and phase inversion composition methods (Anton and Vandamme, 2009; Komaiko and McClements, 2016; Solans and Solé, 2012). As an example, nanoemulsions can be formed using the spontaneous emulsification method by simply mixing a surfactant-oil mixture with water (Anton and Vandamme, 2009; Tadros et al., 2004). The main problems with low-energy methods are that they typically can only be used with synthetic surfactants, a high surfactant-to-oil ratio is required, and the nanoemulsions may breakdown when solution or environmental conditions are changed (Gulotta et al., 2014; Ostertag et al., 2012; Walker et al., 2015).

### High-pressure valve homogenization

**Fig. 4:**
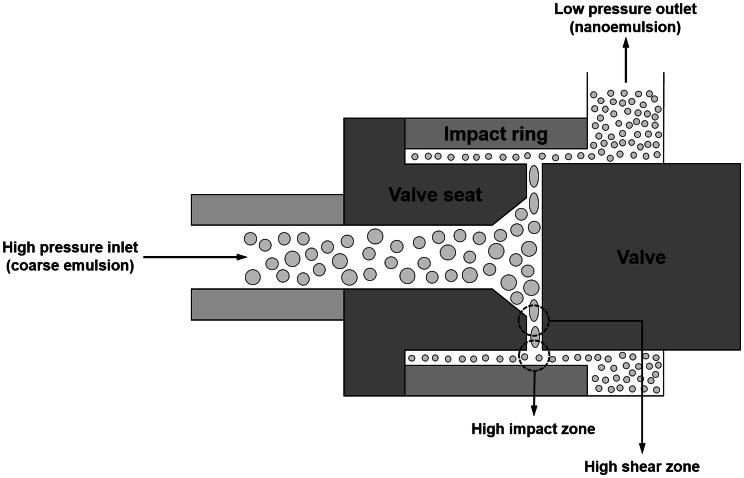
Schematic representation of the size reduction process occurring when coarse emulsions pass through a high-pressure valve homogenizer. The homogenization pressure can be adjusted by varying the gap in the valve

This form of homogenization is commonly used to generate conventional emulsions containing fine droplets in the food industry (Schubert et al., 2003; Schubert and Engel, 2004). It can also be used to form nanoemulsions for certain emulsifier-oil–water systems. Typically, a two-step procedure is used to produce a nanoemulsion. First, a course emulsion premix is formed by blending the oil, water, and emulsifier together using a high shear mixer. Second, this coarse emulsion is forced through a small valve under high-pressure, which leads to the generation of powerful disruptive forces that break the large oil droplets into smaller ones (Fig. 4). Typically, the droplet size in a nanoemulsion produced using a high-pressure valve homogenizer decreases with an increase in number of passes, homogenization pressure, emulsifier concentration, and emulsifier adsorption rate (Mao et al., 2010a; Wang et al., 2008; Yuan et al., 2008b). The oil-to-water viscosity ratio (VR) is also an important parameter affecting the efficiency of droplet disruption using this kind of device. The most efficient droplet disruption, and smallest droplet sizes, are produced when the viscosity ratio is between about 0.05–5 (Tadros et al., 2004; Walstra, 1993). If the oil or aqueous phase is too viscous, then it may be difficult to produce nanoemulsions containing small droplets because of this phenomenon.

### Microfluidization

**Fig. 5:**
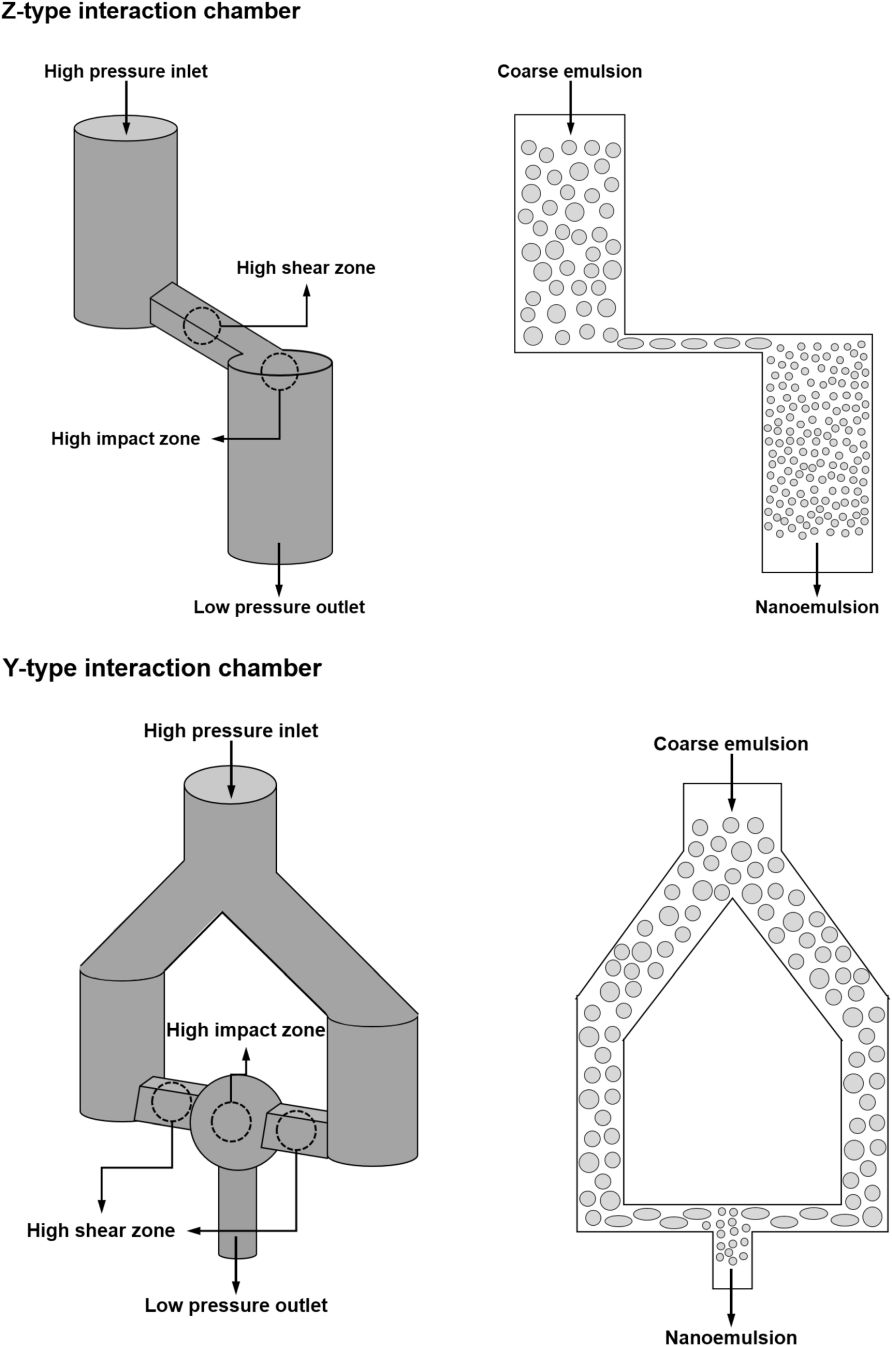
Schematic representation of the Z- and Y-type interaction chambers typically used to produce nanoemulsions using a microfluidizer

Microfluidizers also employ high pressures to break down the large oil droplets in a coarse emulsion into smaller ones (Jafari et al., 2006; Jafari et al., 2007; Schultz et al., 2004). In this case, the coarse emulsion is forced through an inlet channel of an interaction chamber under high pressure. The coarse emulsion is split into two streams that pass through separate channels. The two channels meet at the end of the device, which causes the two streams of emulsion to crash into each other within the interaction chamber (Fig. 5). The collision of the two fast-moving emulsion streams generates intense disruptive forces that efficiently break up the larger droplets into smaller ones. The droplet size produced by microfluidizers tends to decrease with an increase in number of passes, homogenization pressure, emulsifier concentration, and emulsifier adsorption rate (Jafari et al., 2006; Jafari et al., 2007; Leong et al., 2009; Qian and McClements, 2011; Schultz et al., 2004). The oil-to-water viscosity ratio of the system should also be controlled to around unity in order to produce nanoemulsions containing very fine droplets.

### Ultrasonication

Ultrasonicators use high-intensity ultrasonic waves to create nanoemulsions (Jafari et al., 2007; Kentish et al., 2008; Leong et al., 2009; Maa and Hsu, 1999; Schultz et al., 2004). Ultrasonicators can be used to produce nanoemulsions by reducing the size of the droplets in an existing emulsion, or by homogenizing oil, water, and emulsifier together directly. A device capable of generating high-intensity ultrasonic waves (such as a piezoelectric transducer) is used in both batch and flow-through ultrasonicators (Abismaïl et al., 1999; Kentish et al., 2008). The size of the droplets produced by sonication tends to decrease with an increase in sonication time, power level, and emulsifier concentration.

## Lipophilic functional components recently receiving attention

In this section, we examine the properties of some of the most common lipophilic functional ingredients that are encapsulated using nanoemulsion technology. Initially, some of the major challenges with incorporating these ingredients into foods are highlighted.

### Challenge to the incorporation of lipophilic functional components within food products

There are a number of challenges associated with the incorporation of lipophilic functional ingredients into food and beverage products. The relatively poor water-solubility of many of these ingredients means that they cannot be directly dispersed within aqueous-based foods and beverages. This problem can be overcome by encapsulating the functional ingredients within colloidal delivery systems, such as nanoemulsions, solid lipid nanoparticles, microgels, and other types of structured colloids (McClements, 2012a; McClements and Li, 2010; Sagalowicz and Leser, 2010; Velikov and Pelan, 2008). These delivery systems contain colloidal particles that have hydrophobic domains inside them where the lipophilic functional ingredients can be solubilized, as well as hydrophilic exteriors that allow them to be dispersed in water. The low water-solubility of many lipophilic functional ingredients may also negatively impact their oral bioavailability (McClements, 2013). In particular, these ingredients may not be fully absorbed because of their poor solubility in the watery intestinal fluids inside the human gut. In this case, it may be important to use foods that generate colloidal structures (such as mixed micelles) within the gastrointestinal fluids that can solubilize the lipophilic functional ingredients and transport them to the epithelium cells. Many types of bioactive lipophilic ingredients are crystalline at room temperature and therefore must be dissolved or melted prior to homogenization (Fredrick et al., 2010). If they are in a crystalline form within the gastrointestinal tract (GIT), then they may not be efficiently solubilized or absorbed (McClements, 2012b). The vulnerability of some lipophilic functional ingredients to chemical transformation during processing, storage, or digestion may also limit their bioavailability and bioactivity. In this case, it is important to control food composition and environmental conditions to minimize any undesirable chemical changes. Colloidal delivery systems are being specifically designed to increase the water-dispersibility, chemical stability, and bioavailability of lipophilic functional ingredients (McClements, 2010b; Yao et al., 2014). A number of representative lipophilic functional ingredients that might benefit from encapsulation in nanoemulsions are discussed in this section (Table 2).

**Table 2:** Examples of lipophilic nutraceuticals able to have the improved oral bioavailability using nanoemulsion-based delivery systems

Nutraceutical	Molar mass (g/mol)	Melting temperature (°C)	Log*p*
Carotenoid	536.9	187.5	15.5*
α-Carotene	536.9	180.0	15.5*
β-Carotene	552.9	172.0	13.6*
β-Cryptoxanthin	536.9	175.0	15.2*
Lycopene	596.9	182.5	8.2*
Astaxanthin	568.9	196.0	11.8*
Lutein	568.9	215.5	11.8*
Zeaxanthin	368.4	183.0	2.9*
Resveratrol	228.2	255.0	3.0
Coenzyme Q_10_	863.3	49.0	20.9*

If nutraceutical components have values with asterisk (*), values are predicted from their molecular structures

### Lipophilic nutraceuticals

There are a number of lipophilic bioactive molecules that are not essential for human health, but that may be able to enhance health and wellbeing if consumed in sufficiently high levels. These substances are known as nutraceuticals because they are supposed to have some characteristics similar to nutrients (they are naturally found in foods) and some to pharmaceuticals (they may exhibit specific health benefits).

#### Carotenoids

Carotenoids are a diverse group of highly hydrophobic lipophilic molecules that are responsible for the intense red-, orange-, and yellow-colors in many fruits and vegetables, such as carrots, peppers, and tomatoes (Johnson, 2002; Krinsky and Johnson, 2005). Carotenoids are generally divided into two categories: *carotenes*—α-carotene, β-carotene, γ-carotene, and lycopene; and, *xanthophylls*—lutein, zeaxanthin, α-cryptoxanthin, and β-cryptoxanthin (Britton, 1995). The carotenes are only comprised of carbon and hydrogen, whereas the xanthophylls also contain oxygen. Because they contain multiple double bonds, carotenoids can exist in various *trans*/*cis* stereoisomers. Typically, however, most carotenoids are naturally found in the all-*trans* conformation because the *trans* form is more stable than the *cis* form (Guo et al., 2008). Most carotenoids are mainly found in fruits and vegetables, but they may also be found in some edible animal and microbial products (Saini et al., 2015). Carotenoids have a high melting point and therefore are typically found in a crystalline state at ambient temperature, unless they are fully dissolved in an oil phase (Williams et al., 1998). Cooking carotenoid-rich foods with dietary fats is a good way to enhance their bioavailability because the fats can increase the solubility of the carotenoids in the gastrointestinal fluids (Krinsky and Johnson, 2005). Carotenoids have been proposed to demonstrate a range of different beneficial biological activities. First, they are vitamin A precursors that are converted into vitamin A by enzymes in the gastrointestinal tract (Grune et al., 2010). Some well-known examples of provitamin A carotenoids are α-carotene, β-carotene, γ-carotene, and β-cryptoxanthin. These carotenoids may therefore play a similar role in the body as vitamin A, i.e., promote vision, reproduction, and immune systems (Gonçalves et al., 2016; Ribeiro et al., 2011). In addition to their provitamin A activity, many carotenoids are also claimed to have additional health benefits and may therefore be considered to be nutraceuticals. Many caroteonids are effective antioxidants because of their ability to quench singlet oxygen and scavenge free radicals (Fiedor and Burda, 2014). Therefore, carotenoid supplementation could help inhibit cardiovascular diseases or cancers caused by single oxygen and/or free radicals. Carotenoids are easily decomposed by oxygen, light, transition metals, and free radicals because of the numerous conjugated double bonds they contain (Britton, 1995). This is one of main reasons for loss of color and bioactivity (Boon et al., 2010). In spite of the numerous potential health benefits of carotenoids, their low water solubility and chemical instability limit their utilization in many functional food and beverage products (Qian et al., 2012c; Zhang et al., 2016).

#### Curcumin

Ground rhizomes of turmeric (*Curcuma longa*) are used as a spice and pigment in many foods due to their characteristic intense yellow-orange color and flavor profile. Historically, they have also been utilized in many Asian countries as a traditional medicine because of their purported health benefits (Prasad et al., 2014). Recently, turmeric and its major constituents have been extensively studied for their potential application as a bioactive ingredient in functional foods, pharmaceuticals, and cosmetics (Prasad et al., 2014). Curcumin is a polyphenolic phytochemical that is one of the major curcuminoid constituents found within turmeric. Researchers have suggested that curcumin has a number of potential health benefits, including antibacterial, antifungal, anti-inflammatory, antioxidant, antitumor, and antiviral activities (Kocaadam and Şanlier, 2017). Functional foods and beverages fortified with curcumin have recently attracted considerable attention because of their potential to improve human health and well-being through diet. The application of curcumin as a nutraceutical agent in foods is, however, currently limited because of its poor water-solubility, high melting point, and chemical instability (Anand et al., 2007).

#### Resveratrol

Resveratrol is a natural polyphenol that is part of the stilbenoid family that is mainly found in plants, such as mulberries, peanuts, and grapes (particularly the seeds) (Burns et al., 2002). It has become of great interest to the food and pharmaceutical industries because of its potential health beneficial effects, including anticarcinogenic, anti-inflammatory, anti-obesity, antioxidant, and cardioprotective effects (King et al., 2006; Smoliga et al., 2011). The fortification of foods with resveratrol is often difficult because of its low water-solubility, chemical instability, and low bioavailability (Wenzel and Somoza, 2005). Resveratrol may exist in either a *cis* or a *trans* forms, but only the *trans* form has biological activity. Unfortunately, the bioactivity of resveratrol may be reduced due to chemical degradation or isomerization induced by exposure to light, certain pH values, and heat.

#### Coenzyme Q_10_

Coenzyme Q substances (also known as ubiquinone) are part of the benzoquinone family, and are lipid-soluble bioactive compounds (Ernster and Dallner, 1995). The predominant form of this bioactive substance in animals and humans is coenzyme Q_10_ (CoQ_10_), which contains 10 isoprenoid units as a hydrophobic side chain. CoQ_10_ is an essential component in mitochondria energy metabolism of all living cells, because it participates in energy conversion from carbohydrates and fatty acids into adenosine triphosphate (ATP) (Crane, 2007; Pravst et al., 2010). Recent studies show that Coenzyme Q_10_ is a very powerful antioxidant that is able to scavenge free radicals generated under oxidative stresses in the human body (Bentinger et al., 2007; Petillo and Hultin, 2008). Ubiquinol (CoQ_10_H_2_) has been reported to have a higher antioxidant activity than the other two redox states (fully oxidized ubiquinone (CoQ_10_) and partially reduced ubisemiquinone (CoQ10^**·**−^) (Frei et al., 1990). CoQ10 deficiency has been linked with increased risk of cancer (Bank et al., 2011), cardiovascular disease (Singh et al., 2007), Parkinson’s disease (Mancuso et al., 2010), and type 2 diabetes (Salles et al., 2006). Consequently, there has been considerable interest in using CoQ_10_ as a drug or dietary supplement (Pravst et al., 2010). Like the other lipophilic nutraceuticals discussed earlier, the incorporation of CoQ_10_, into foods is often limited by its low water-solubility, chemical instability, and poor bioavailability. Previous studies have shown that these problems can be overcome using emulsion-based delivery systems (Paroha et al., 2018). CoQ10 has also been added to food products to extend their shelf life because of its ability to inhibit lipid oxidation (Qian et al., 2012a; Zhao et al., 2013).

### Oral bioavailability of lipophilic functional components

For the bioactive ingredients in food products to maintain their health promoting effects, they have to remain stable during processing, storage, and transportation. After ingestion, they must be released from the food matrix, stable within the gastrointestinal fluids, absorbed through the epithelium cells, and then reach the systemic circulation where they can exhibit their biological activity (Singh et al., 2015). The oral bioavailability of a bioactive ingredient is typically defined as the fraction reaching the systemic circulation (blood or lymph) in an active form (Parada and Aguilera, 2007). The oral bioavailability (*F*) of a functional component can be described by the following expression (Aungst, 2017; Yao et al., 2015):

6 $$F = F_{\text{B}} \times F_{\text{A}} \times F_{\text{M}}$$

Here, *F*_B_ is the fraction of the bioactive that is present in a *bioaccessible* form, *F*_A_ is the fraction that is *absorbed* by the epithelium cells, and *F*_M_ is the fraction that enters the systemic circulation in an active form after being exposed to any *metabolic* processes. The rate limiting factor determining the overall bioavailability of a bioactive agent depends on its molecular characteristics, as well as the nature of the surrounding food matrix.

#### Bioaccessibility of lipophilic functional components

For a lipophilic substance, the bioaccessibility depends on its liberation from the food matrix and then its solubilization in the gastrointestinal fluids. The food matrix may be disrupted and the lipophilic ingredients released during the passage of a food through the upper GIT, where it is exposed to saliva, digestive enzymes, bile salts, pH changes, and mechanical forces. Chewing in the mouth, churning in the stomach, and peristalsis in the intestine help break down the food matrix. After release, lipophilic substances usually have to solubilized within the aqueous gastrointestinal fluids before being transport to the intestinal epithelial cells. The solubility of these substances is therefore one of the most important factors impacting their overall bioavailability (Buckley et al., 2013). During digestion, lipophilic substances are solubilized within the mixed micelles formed from bile salts, phospholipids, and lipid digestion products (Yao et al., 2014; Yao et al., 2015). The most important lipid digestion products are monoacylglycerols and free fatty acids resulting from the action of lipase on ingested triacylglycerols. The bioaccessibility of lipophilic substances depends on the solubilization capacity of the mixed micelles in the small intestine, which in turn depends on the type and amount of digestible lipids consumed (McClements and Xiao, 2014; Porter et al., 2007).

#### Absorption of lipophilic functional components

In general, lipophilic substances may be absorbed by epithelial cells through various passive or active transport mechanisms (Yao et al., 2014). The precise absorption mechanism for a particular lipophilic substance depends on its molecular properties, as well as the nature of the surrounding food matrix. (McClements and Xiao, 2014). At present, there is a relatively poor understanding of the absorption mechanism involved for different kinds of bioactive agents and foods.

#### Metabolism of lipophilic functional components

Lipophilic substances may be metabolized within the human gut or after they have been absorbed and then distributed among the different organs inside the human body (McClements, 2015a; McClements and Xiao, 2014). In the case of lipids, short- and medium-chain fatty acids typically go to the liver through the portal vein while long chain fatty acids go through the lymph system (McClements, 2010a; Porter et al., 2007; Stipanuk and Caudill, 2012). Consequently, the hydrophobicity of lipophilic substances determines how they are distributed within the human body. Nutraceuticals with a high hydrophobicity would be expected to go through the lymphatic route and be carried around the body in lipoproteins (such as chylomicrons), but nutraceuticals with a low hydrophobicity would be expected to go to the liver. The molecular structures and biological activities of lipophilic substances can be greatly altered by metabolic processes within different regions of the human body, particularly the liver (McClements and Xiao, 2014).

## Nanoemulsion applications: encapsulating and improving bioavailability of lipophilic functional components

This section provides an overview of studies that have used nanoemulsions to encapsulate lipophilic functional components and increase their bioavailability. Nanoemulsion-based delivery systems have been created using both high- and low-energy methods. As mentioned earlier, however, low-energy methods are not suitable for many food applications because using high levels of synthetic surfactants to formulate nanoemulsions could lead to taste, health, or economic concerns (Öztürk, 2017; Walia et al., 2019; Yang et al., 2012). For this reason, this review mainly focuses on the research on nanoemulsions produced using high-energy methods.

### Nanoemulsions for lipophilic nutraceuticals

In this section, the utilization of nanoemulsions to encapsulate a number of lipophilic nutraceuticals is reviewed. The utilization of nanoemulsions to encapsulate and deliver fat-soluble vitamins has recently been reviewed in detail elsewhere and so the reader is referred to this work (Öztürk, 2017).

#### Carotenoids

Recently, there have been numerous studies on the encapsulation of carotenoids using nanoemulsions and other colloidal delivery systems (Dos Santos et al., 2018).

##### Carotenes

Many researchers have focused on optimization of the conditions employed to prepared β-carotene nanoemulsions using high energy methods. In the studies of Jo and Kwon (2014), Mao et al. (2010a; 2010b), Yuan et al. (2008a), and Yuan et al. (2008b), the effects of system composition and homogenization method on nanoemulsion formation, stability, and properties were examined. In particular, the impact of β-carotene concentration, emulsifier type, and emulsifier concentration were investigated, as well as the impact of homogenizer type and homogenization conditions (temperature, pressure, and number of passes). Luo et al. (2017) fabricated β-carotene-nanoemulsions by dual-channel microfluidization using quillaja saponins and whey protein isolate as emulsifiers. They evaluated the effects of emulsifier concentration and microfluidization pressure on the formation and stability of these nanoemulsions. All nanoemulsions, independent of emulsifier type, were physically stable during storage at 4, 25, or 55 °C for 14 days. Also, Luo et al. (2017) reported that the β-carotene degradation rate did not depend on emulsifier type used for dual-channel microfluidization. Silva et al. (2011) prepared β-carotene-nanoemulsions using a high-energy emulsification-evaporation technique. They studied the effects of homogenization time, shear rate, and number of passes on the droplet size of the nanoemulsions. The mean droplet diameter (*d*_4,3_) of the β-carotene-nanoemulsions prepared using this approach ranged from 9 to 280 nm, which suggests that in some cases microemulsions or micelles were formed. These researchers reported that the degree of oxygenation of the nanoemulsions during processing played a major role in determining the chemical stability of the β-carotene in the nanoemulsions. In another study, Kim et al. (2014) optimized the conditions (number of passes, lycopene concentration, and emulsifier concentration) required to produce lycopene-loaded nanoemulsions using a homogenization-evaporation process.

Qian et al. (2012b) studied the impact of carrier oil type (long-chain triglyceride, medium-chain triglyceride, or orange oil) on the droplet size and bioaccessibility of β-carotene-nanoemulsions. Independent of carrier oil type, they could create nanoemulsions containing oil droplets < 200 nm in diameter. The bioaccessibility of β-carotene was much higher in the nanoemulsions prepared with long-chain triglyceride than in the ones prepared with medium-chain triglycerides. This effect was attributed to the fact that the mixed micelles formed from the digestion products of the long-chain triglyceride had larger hydrophobic domains to accommodate the highly hydrophobic carotenoids. They also reported that the bioaccessibility of β-carotene was very low in orange oil nanoemulsions, which was attributed to the fact that this kind of oil does not form mixed micelles. These findings were consistent with the results of Rao et al. (2013) who studied the effects of carrier oil composition on β-carotene bioaccessibility in nanoemulsions prepared from corn oil, lemon oil, and their mixtures. In another study, Yi et al. (2015) reported that there was no significant difference in the mean droplet diameter of β-carotene-nanoemulsions prepared from a range of carrier oils, including medium-chain triglycerides, coconut oil, palm oil, olive oil, canola oil, and corn oil. However, the bioaccessibility of the carotenoids in the nanoemulsions was again reported to be better for the long chain triglycerides. Yi et al. (2015) reported that nanoemulsions formulated using unsaturated fatty acid-rich lipids led to a greater β-carotene bioaccessibility than those formulated from saturated fatty acid-rich ones. Salvia-Trujillo et al. (2013b) also studied the effect of oil composition (medium-chain triglycerides, long-chain triglycerides, and their mixtures) on the bioaccessibility of β-carotene encapsulated in nanoemulsions using a simulated gastrointestinal process. The total amount of free fatty acids produced after digestion increased as the long-chain triglyceride to medium-chain triglyceride ratio of the lipid phase increased, but the bioaccessibility of β-carotene decreased. Zhang et al. (2016) studied the effect of oil phase composition (long-chain triglycerides, medium-chain glycerides, orange oil, and their mixtures) on the bioaccessibility of β-carotene encapsulated in nanoemulsions formed by spontaneous emulsification. Their results were consistent with the results of previous studies on similar nanoemulsions produced by high-energy methods (Qian et al., 2012b).

Salvia-Trujillo et al. (2013a) assessed the influence of droplet size on the bioaccessibility of β-carotene by preparing emulsions having small (*d*_4,3_ = 0.2 μm), medium (*d*_4,3_ = 0.4 μm), and large droplets (*d*_4,3_ = 23.0 μm) using Tween 20 and corn oil and a microfluidizer. As the mean droplet diameter of the emulsions decreased, the bioaccessibility of the β-carotene increased. This effect was attributed to the fact that not all of the large droplets were digested by the end of the small intestine phase. As a result, not all of the β-carotene was released from the oil droplets, and there were less mixed micelles available to solubilize the β-carotene that was released.

In the study of Qian et al. (2012c), the influence of temperature, pH, and ionic strength on the physical and chemical stability of β-carotene-loaded nanoemulsions was investigated by preparing them with β-lactoglobulin by microfluidization. Ionic strength (0–500 mM of NaCl) did not significantly affect β-carotene degradation but storing at elevated temperatures did. In addition, β-carotene degradation was faster under highly acidic conditions (pH 3) than under weakly acidic, neutral, or weakly basic conditions (pH 4–8).

Liang et al. (2013) studied the effect of the molecular density of OSA-starches on the bioaccessibility of β-carotene in nanoemulsions stored at 25 °C in the dark for 30 days. They found that the bioaccessibility was slightly higher when a high molecular density OSA-starch was used (69%) than when a low molecular density one was used (62%). This effect could be due to differences in the thickness and density of the interfacial films formed around the oil droplets in the nanoemulsions. Zhang et al. (2017) evaluated the impact of pH, ionic strength, temperature, and storage time on the stability of β-carotene-nanoemulsions formed using sodium caseinate-stabilized as an emulsifier.

The ability of antioxidants to inhibit β-carotene degradation in nanoemulsions was shown to be dependent on their solubility characteristics (Qian et al., 2012a; Yi et al., 2016). Generally, oil-soluble antioxidants were better at inhibiting β-carotene degradation in nanoemulsions than water-soluble ones.

##### Xanthophylls

Compared to carotenes, there is much less research on the encapsulation and protection of xanthophylls in nanoemulsions. In the study of Liu et al. (2018), the bioaccessibility of astaxanthin encapsulated in nanoemulsions was evaluated using a simulated gastrointestinal tract. These nanoemulsions were prepared using different kinds of long-chain triglycerides (flaxseed, olive, and corn oil) by microfluidization. The final amount of free fatty acids released and astaxanthin bioaccessibility (respectively) decreased in the following order: olive oil (87 and 68%) > flaxseed oil (81.5 and 53%) > corn oil (74.5 and 48%). Salvia-Trujillo et al. (2015) evaluated the bioavailability of fucoxanthin encapsulated in nanoemulsions formulated using different carrier oils (long-chain triglycerides, medium-chain triglycerides, and orange oil) by microfluidization. The bioaccessibility of fucoxanthin determined using a simulated gastrointestinal tract model was higher in the nanoemulsions prepared from the digestible oils than in the ones prepared with the indigestible oils for the reasons discussed earlier. Rat feeding studies revealed that the amount of fucoxanthin absorbed by the intestinal epithelia cells was higher in nanoemulsions prepared with long- and medium-chain triglycerides than in ones prepared with orange oil. However, the concentration of fucoxanthin in the serum of the rats was similar independent of the digestibility of the carrier oils.

#### Curcumin

Ahmed et al. (2012) examined the influence of lipid phase composition on the bioaccessibility of curcumin encapsulated within nanoemulsions. In this study, the nanoemulsions were prepared using long-, medium-, or short-chain triglycerides as the carrier oil by high pressure homogenization. The nanoemulsions prepared with long- and medium-chain triglycerides contained droplets having relatively small diameters (181 and 174, respectively) with narrow droplet size distributions. However, the nanoemulsions prepared with short-chain triglycerides contained relatively large droplets (≈ 2,000 nm), which was attributed to rapid Ostwald ripening. The total amount of free fatty acids produced at the end of digestion decreased in the order: medium-chain > short-chain > long-chain triglycerides, while the initial rate of lipid digestion decreased in the order: short-chain > medium-chain > long-chain triglycerides. The bioaccessibility of curcumin decreased in the order: medium-chain > long-chain > short-chain triglycerides. The findings of Ahmed et al. (2012) indicated that the type of lipid used to form nanoemulsions might have a considerable influence on curcumin encapsulation and bioaccessibility.

Sari et al. (2015) encapsulated curcumin within nanoemulsions formulated using medium-chain triglycerides as an oil phase and whey protein or Tween 80 as emulsifiers using sonication. The nanoemulsions had mean droplet diameters of around 141 nm and curcumin encapsulation efficiencies of around 91%. Simulated gastrointestinal tract studies showed that the curcumin-enriched nanoemulsion were relatively resistant to digestion in the stomach, but slowly released curcumin during digestion in the small intestine. They also found that the encapsulation of curcumin within the nanoemulsions reduced its antioxidant activity.

Chuacharoen and Sabliov (2019) compared the ability of nanoemulsions and biopolymer nanoparticles to prevent the chemical degradation of curcumin within a model food matrix during processing and storage. They prepared nanoemulsions (medium-chain triglycerides) and biopolymer nanoparticles (zein) using the same type and level of emulsifiers (lecithin and Tween 80). The biopolymer nanoparticles were more effective at protecting the curcumin from degradation under environmental stresses (such as pH and temperature) than the nanoemulsions. However, long-term storage studies showed that the curcumin trapped into the nanoemulsion was retained more effectively than in the nanoparticles. Kumar et al. (2016) also tested the ability of the nanoemulsions stabilized with sodium caseinate to protect curcumin when exposed to pH, ionic strength, and temperature variations.

In the study of Li et al. (2016), nanoemulsions composed of medium-chain triglycerides, Tween 80, and lecithin prepared by sonication were shown to have a high curcumin loading capacity and encapsulation efficiency. The used of chitosan as a coating agent increased the stability of the encapsulated curcumin against thermal and UV-mediated degradation. Joung et al. (2016) showed that the oil-to-emulsifier-to-water ratio affected the size of the droplets produced in curcumin-loaded nanoemulsions. Curcumin-loaded nanoemulsion prepared under optimum conditions were shown to be physically stable for 30 days at both 4 and 25 °C.

Zou et al. (2016) tested the effect of the original curcumin location in nanoemulsions on curcumin bioaccessibility. In this study, they prepared curcumin-rich nanoemulsions by adding curcumin in the corn oil before homogenization or by mixing curcumin with an already prepared nanoemulsion. They found that when curcumin was added to the system did not greatly affect its bioaccessibility. Zou et al. (2016) also evaluated the effect of protein addition on the bioaccessibility of curcumin in the nanoemulsions. They observed that protein addition did not significantly affect the bioavailability of curcumin. The effect of protein (bovine serum albumin) on the physicochemical properties of curcumin-enriched nanoemulsion prepared by sonication was also studied by Kaur et al. (2015).

To improve the water-dispersibility of curcumin, Moghaddasi et al. (2018) studied the effect of oil-to-emulsifier ratio and water-to-oil ratio on the droplet size of curcumin-loaded colloidal dispersions prepared using black pepper oil by spontaneous emulsification. Under optimal conditions (5:1 water/oil, 1:9 black pepper oil/Tween 80), the mean particle size was approximately 16 nm, which suggests that microemulsions (rather than nanoemulsions) were formed. Curcumin encapsulated within the colloidal delivery systems exhibited higher antioxidant activity than a suspension of curcumin crystals dispersed in water.

A number of researchers have examined the possibility of using structural design principles to create more sophisticated colloidal delivery systems. Zeeb et al. (2015) prepared curcumin-loaded nanoemulsions using either spontaneous emulsification or microfluidization, and then encapsulated the nutraceutical-loaded droplets within filled hydrogel beads. Abbas et al. (2015) fabricated multilayered nanoemulsions using chitosan (cationic) and carboxymethyl cellulose (anionic) to coat the oil droplets in curcumin-loaded nanoemulsions prepared from OSA-starch by sonication. Yu and Huang (2012) investigated the possibility of using organogel-based nanoemulsions as delivery systems for curcumin. Several other studies have used nanoemulsions to encapsulate turmeric extract rather than curcumin (Foujdar et al., 2018; Hong et al., 2019; Park et al., 2019).

#### Resveratrol

Kumar et al. (2017) studied the potential of using nanoemulsion-based delivery systems to encapsulate resveratrol. Resveratrol-loaded nanoemulsions were prepared using a mixture of lecithin and Tween 80 as emulsifiers, and a sonicator as a homogenizer. The mean droplet diameter of the nanoemulsions was 20 nm, which again suggests that they might have been microemulsions. The loading of the resveratrol in the nanoemulsions was relatively high (> 99%), while the cumulative drug release was approximately 55%. Resveratrol encapsulated within nanoemulsions had better chemical stability to UV-light irradiation than resveratrol dispersed in ethanol or water.

The effect of emulsifiers on the formation, stability, and functionality of resveratrol-loaded nanoemulsions was studied by preparing them from soy lecithin, sucrose palmitate, and glycerol monooleate using a high-pressure homogenizer (Sessa et al., 2011). During 30 days of storage at 55 °C, the mean droplet diameter of the nanoemulsions prepared from either soy lecithin or soy lecithin/sucrose palmitate remained relatively constant, but it increased appreciably for ones prepared from Tween 20/glycerol monooleate. The nanoemulsions prepared with soy lecithin/sucrose palmitate and Tween 20/glycerol monooleate inhibited the isomerization and degradation of resveratrol during UV-light exposure. FRAP and ORAC assays showed that nanoemulsions formulated using soy lecithin/sucrose palmitate and Tween 20/glycerol monooleate were able to inhibit the loss of antioxidant activity of resveratrol during in vitro gastrointestinal digestion. The bioavailability of resveratrol in nanoemulsions prepared with soy lecithin, sucrose palmitate, and glycerol monooleate was also investigated by assessing the sustained release and permeation through simulated intestinal cell walls using Caco-2 cell monolayers (Sessa et al., 2014). These researchers showed that resveratrol encapsulated within nanoemulsions prepared with soy lecithin was transported through the Caco-2 cell monolayers much more effectively than within nanoemulsions prepared with soy lecithin/sucrose palmitate or Tween 20/glycerol monooleate.

Spigno et al. (2013) formulated nanoemulsions containing red-grape marc extract (which contains high levels of resveratrol) from sunflower oil and soy lecithin using high pressure homogenization. These nanoemulsions were intended to be used as a natural preserving agent in hazelnut paste. The nutraceutical-loaded nanoemulsions were shown to inhibit lipid oxidation in the hazelnut paste.

The possibility to producing resveratrol-enriched nanoemulsions using coconut oil and soybean lecithin was evaluated by Hung et al. (2006). The researchers showed that lecithin-stabilized coconut oil nanoemulsions could be prepared, but the encapsulation and retention of the resveratrol during storage was not successful. The authors proposed that this could have been due to the crystallization of the coconut oil (its melting point ranges from 23 to 27 °C) during storage, which would result in the expulsion of the resveratrol from the oil droplets.

To evaluate the effect of droplet size on the chemical stability of encapsulated resveratrol, Davidov-Pardo and McClements (2015) prepared nanoemulsions with different mean droplet diameters (220, 99, and 45 nm) using spontaneous emulsification or microfluidization. After exposure to UV-light for 60 min, almost 90% of the resveratrol remained in the nanoemulsions with mean droplet diameters of 220 nm but only about 70% remained in the nanoemulsions with mean droplet diameters of 45 nm. This result was attributed to the fact that UV light could penetrate into the nanoemulsions with the smaller droplets more easily. The researchers optimized the nanoemulsion formulation (oil composition and surfactant-to-oil ratio) to obtain small resveratrol-loaded oil droplets with good stability to growth.

To improve the oral bioavailability of resveratrol, Mamadou et al. (2017) evaluated the possibility of producing resveratrol-enriched liquid and semi-solid nanoemulsions by self-emulsification (a low-energy method). The liquid nanoemulsions were formed from medium-chain triglycerides and Tween 80, while the semi-solid nanoemulsions were formed from lauroyl polyoxyl-32 glycerides and caprylocaproyl polyoxyl-8 glycerides. These two types of nanoemulsions considerably increased the intestinal permeation of resveratrol across the jejunum.

#### Coenzyme Q_10_

A number of researchers have been conducted on the design of nanoemulsions for the encapsulation, protection, and release of CoQ_10_. Zhou et al. (2014a; 2014b) prepared CoQ_10_-enriched nanoemulsions stabilized with D-α-polyethylene glycol 1000 succinate, polyoxyl 40 hydrogenated castor oil, polyglycerol 10 stearic acid ester, and sucrose palmitate using a high-pressure homogenizer at 60 °C. They then evaluated the bioavailability of CoQ_10_ after oral administration in rats. They demonstrated that nanoemulsions could improve the oral bioavailability of CoQ_10_ compared with a simple CoQ_10_-suspension.

CoQ_10_-loaded nanoemulsions have also been formulated using extra virgin olive or olive–pomace oil using Tween 20 or Tween 40 as emulsifiers and a sonicator as a homogenizer (Katsouli and Tzia, 2019). These nanoemulsions had good physical stability and the amount of CoQ_10_ remaining in them was still around 50% after 90 days of storage. Nanoemulsions prepared with extra virgin olive oil had better physical stability and CoQ_10_ retention than those prepared with olive–pomace oil. Belhaj et al. (2012) evaluated the effect of fatty acid composition of carrier oils on the bioavailability of CoQ_10_ in nanoemulsions formed using a high-pressure homogenizer. The bioavailability of CoQ_10_ in nanoemulsions prepared from salmon oil, which is rich in polyunsaturated fatty acids, was twofold higher than from nanoemulsions prepared from soybean oil, which is rich in monounsaturated fatty acids.

Oral administration of CoQ_10_-loaded nanoemulsions formulated from either digestible (corn oil) or indigestible (mineral oil) oils was conducted (Cho et al., 2014). The bioavailability of CoQ_10_ was higher in the nanoemulsions prepared from the digestible oil than from the indigestible oil, highlighting the importance of selecting an appropriate carrier oil type when formulating nanoemulsions.

Cheuk et al. (2015) showed that CoQ_10_-enriched nanoemulsions could be produced with OSA-starch using a high-pressure homogenizer that had mean droplet diameters around 250 nm. After freeze-drying and reconstitution, there was little change in the particle size and a high retention of CoQ_10_ (98.2%).

The pharmacokinetic parameters and bioavailability of CoQ_10_ encapsulated in nanoemulsions prepared using spontaneous emulsification was compared to those of CoQ_10_ crystal suspensions using in vivo tests in rats (Hatanaka et al., 2008). Similar *T*_max_ values (time to maximum plasma CoQ_10_ concentration) was around 1.5 and 1.7 h for CoQ_10_-enriched nanoemulsions and CoQ_10_ crystal suspensions, but the nanoemulsion had about 1.7-fold higher AUC (area under the curve of plasma CoQ_10_ concentration *versus* time) and *C*_max_ (maximum plasma CoQ_10_ concentration) than the crystal suspension. CoQ_10_-enriched nanoemulsions have also been prepared from a blend of sunflower oil and Tween 20 using spontaneous emulsification (Chen et al., 2015). This study suggested that the use of synthetic emulsifiers could be reduced for CoQ_10_-enriched nanoemulsion formation through a careful blending of natural and synthetic emulsifiers.

## Conclusions

Nanoemulsions have advantages over conventional emulsions for certain applications because of the unique physicochemical and functional characteristics associated with their small droplet size. As shown in this article, a variety of lipophilic nutraceuticals can be encapsulated within nanoemulsions, which can increase their water-dispersibility, stability, and bioavailability. The nanoemulsions, however, have to be carefully formulated. In particular, lipid phase composition, interfacial properties, and particle size have to be controlled to obtain appropriate functional attributes. This can be achieved by controlling nanoemulsion composition and homogenization conditions. Both in vitro and in vivo studies have shown that the oral bioavailability of lipophilic nutraceuticals can be increased substantially using nanoemulsion technology.
