# Correction to: Nanoemulsions as delivery systems for lipophilic nutraceuticals: strategies for improving their formulation, stability, functionality and bioavailability

**DOI:** 10.1007/s10068-020-00785-9

**Published:** 2020-07-08

**Authors:** Seung Jun Choi, David Julian McClements

**Affiliations:** 1 Department of Food Science and Technology, Seoul National University of Science and Technology, Seoul, 01811 Republic of Korea; 2 Departement of Interdisciplinary Bio IT Materials, Seoul National University of Science and Technology, Seoul, 01811 Republic of Korea; 3 Department of Food Science, University of Massachusetts, Amherst, MA 01003 USA; 4 Department of Food Science and Bioengineering, Zhejiang Gongshang University, Hangzhou, 310018 Zhejiang China

## Correction to: Food Sci Biotechnol (2020) 29(2):149–168 10.1007/s10068-019-00731-4

The article “Nanoemulsions as delivery systems for lipophilic nutraceuticals: strategies for improving their formulation, stability, functionality and bioavailability”, written by Seung Jun Choi and David Julian McClements, was originally published Online First without Open Access. After publication in volume 29 issue 2, page 149–168 the author decided to opt for Open Choice and to make the article an Open Access publication. Therefore, the copyright of the article has been changed to © The Author(s) 2020 and the article is forthwith distributed under the terms of the Creative Commons Attribution 4.0 International License (http://creativecommons.org/licenses/by/4.0/), which permits use, duplication, adaptation, distribution and reproduction in any medium or format, as long as you give appropriate credit to the original author(s) and the source, provide a link to the Creative Commons license, and indicate if changes were made.

The original article has been corrected.

